# Deceptively Simple yet Profoundly Impactful: Text Messaging Interventions to Support Health

**DOI:** 10.2196/58726

**Published:** 2024-08-27

**Authors:** Brian Suffoletto

**Affiliations:** 1 Department of Emergency Medicine Stanford University Palo Alto, CA United States

**Keywords:** SMS intervention, behavior, intervention, review, text messaging, SMS, interventions, behaviors, behaviour, behaviours, effectiveness, development, impact, narrative review, physical activity, diet, weight loss, mental health, substance use, meta-analysis, chatbot, chatbots, large language model, LLM, large language models, mobile phone

## Abstract

This paper examines the use of text message (SMS) interventions for health-related behavioral support. It first outlines the historical progress in SMS intervention research publications and the variety of funds from US government agencies. A narrative review follows, highlighting the effectiveness of SMS interventions in key health areas, such as physical activity, diet and weight loss, mental health, and substance use, based on published meta-analyses. It then outlines advantages of text messaging compared to other digital modalities, including the real-time capability to collect information and deliver microdoses of intervention support. Crucial design elements are proposed to optimize effectiveness and longitudinal engagement across communication strategies, psychological foundations, and behavior change tactics. We then discuss advanced functionalities, such as the potential for generative artificial intelligence to improve user interaction. Finally, major challenges to implementation are highlighted, including the absence of a dedicated commercial platform, privacy and security concerns with SMS technology, difficulties integrating SMS interventions with medical informatics systems, and concerns about user engagement. Proposed solutions aim to facilitate the broader application and effectiveness of SMS interventions. Our hope is that these insights can assist researchers and practitioners in using SMS interventions to improve health outcomes and reducing disparities.

## Introduction

SMS text messaging interventions have emerged as a significant tool in the landscape of digital health, bridging the expanding gap between health care provider constraints and patient needs. While deceptively simple in design, SMS interventions are grounded in a sophisticated blend of communication strategies, psychological insights, and technological advances, all of which have been shown to positively shape health behaviors. This paper aims to provide a comprehensive overview of the SMS interventions’ trajectory, distilling the evidence of their impact, discussing the critical elements of successful interventions, and exploring future directions for research and application.

Our examination begins with a retrospection of the emergence and evolution of SMS interventions, starting from the early 2000s when digital communication first showed promise for supporting health behavior change. Our paper highlights the broad scope of funding from US government agencies and the expanding research in this field. A narrative review then spotlights the success of SMS interventions across critical health domains—physical activity, diet and weight loss, mental health, and substance use—drawing from recent meta-analyses. The paper explores the specific aspects of SMS interventions that contribute to their effectiveness with a focus on the real-time capability of SMS interventions to collect information and deliver microdoses of intervention support.

The paper then discusses crucial SMS intervention design considerations across categories of communication strategies, psychological underpinnings, behavior change techniques (BCTs), and enhanced functionalities. It also identifies key unanswered questions that future research needs to address. It then discusses the evolution of SMS interventions from rule-based systems (eg, branching algorithms) to generative artificial intelligence systems. It focuses on recent advancements in large language models (LLMs), which present new opportunities for enhancing the interactivity and personalization of SMS interventions, promising to elevate the user experience and engagement further.

Finally, the paper discusses existing SMS intervention implementation challenges, including transitioning SMS interventions from research prototypes to widely implemented health interventions, the absence of a centralized platform for dissemination akin to app stores, legal and ethical concerns around data security and patient privacy, and the intricacies of integrating SMS interventions with existing electronic health record systems. The paper then offers suggestions for overcoming such barriers by combining innovation in technology development, policy formulation, and practice implementation.

Our goal was to equip researchers, health care practitioners, and policy makers with knowledge on how to effectively use SMS interventions, aiming to improve health outcomes and reduce health care access and quality disparities. By offering a thorough analysis and forward-looking perspective, this paper contributes to the broader conversation on SMS interventions’ role in health promotion and disease prevention.

## Growth of SMS Intervention Scholarship and Funding

**Figure 1:**
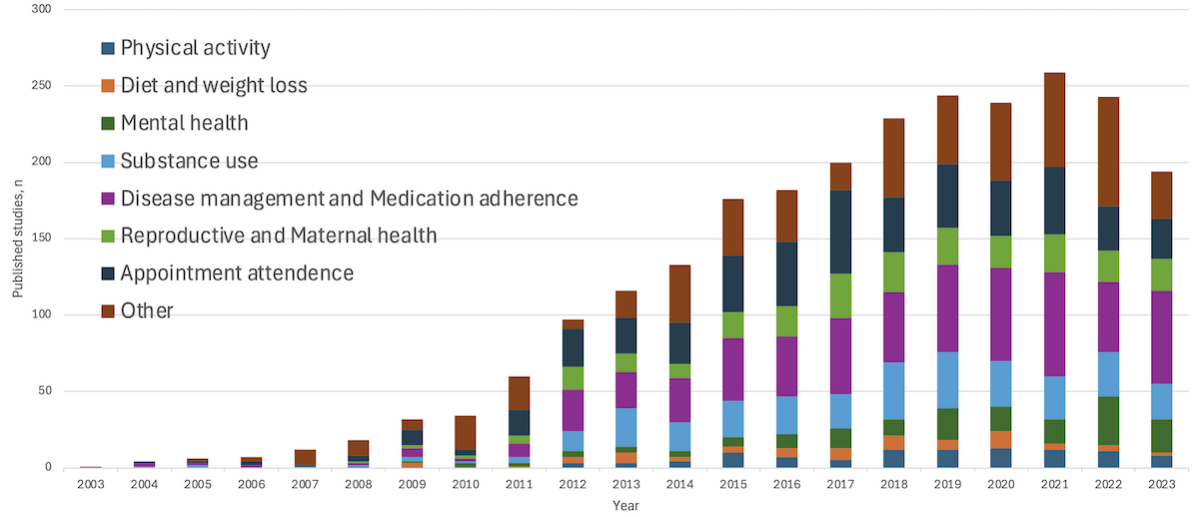
Number of published peer-reviewed studies of SMS interventions by health-related categories and year.

To identify peer-reviewed publications on SMS interventions across key health-related categories, we conducted a comprehensive search on PubMed. This search included categories of physical activity, diet and weight loss, mental health, substance use, disease management, and medication adherence. Multimedia Appendix 1 lists the PubMed search terms. As shown in Figure 1, the history of SMS interventions for behavioral support starts in the early 2000s and appears to peak in 2021, with >250 peer-reviewed papers published that year. The data show growth across key health-related categories. The most substantial growth was observed in the “disease management and medication adherence” category, underscoring the perceived efficacy and value of SMS interventions in addressing daily health-related behaviors. While the number of studies in some categories, such as physical activity and diet and weight loss, has remained relatively low, there has been a consistent increase in research on mental health, substance use, reproductive and maternal health, and appointment attendance using SMS interventions. Conversely, the relatively slower increase in research on physical activity and diet and weight loss may reflect the complexities of influencing lifestyle behaviors through digital means alone or the saturation of research in these areas.

To identify US national funding trends for SMS intervention projects, we queried the National Institutes of Health Reporter website using the search term “text message AND intervention.” We report the diversity of SMS intervention projects from 2007 to 2023 from the top 16 divisions of NHI. The National Cancer Institute (NCI) leads with 312 projects, followed by the National Institute of Mental Health with 274 projects and the National Institute on Drug Abuse with 239 projects. Other notable institutes include the National Institute of Diabetes and Digestive and Kidney Diseases; the National Heart, Lung, and Blood Institute; the Eunice Kennedy Shriver National Institute of Child Health and Human Development; and the National Institute on Minority Health and Health Disparities, all administering >170 projects each.

The major themes of NCI funding for SMS interventions to date include smoking cessation for specific groups such as pregnant women, screening for cervical and colorectal cancers in underserved areas, improvement of adherence to cancer treatments, and cancer prevention initiatives such as human papillomavirus vaccination. The National Institute of Mental Health has focused on SMS interventions for postpsychiatric emergency care, HIV-related health behaviors, and mental health condition monitoring. The National Institute on Drug Abuse has targeted funding at managing substance use disorders, emphasizing treatment engagement and recovery support. The National Institute of Diabetes and Digestive and Kidney Diseases funded SMS interventions aimed at diabetes management, obesity prevention, and kidney disease care. The National Heart, Lung, and Blood Institute invested in SMS interventions for medication adherence in sickle cell disease, hypertension and asthma management, and lifestyle modifications to reduce cardiovascular risk. Finally, the National Institute on Minority Health and Health Disparities supported a wide array of SMS interventions addressing health disparities, including SMS interventions for enhancing cancer screening attendance, obesity prevention in Hispanic women, health optimization in taxi drivers, workplace inclusion, physical activity in African American cancer survivors, sugary beverage reduction, maximizing COVID-19 vaccination uptake, and smoking cessation among immigrant populations.

## Narrative Review of SMS Interventions Across Key Health-Related Categories

### Overview

In this section, we review recent meta-analyses on the impact of SMS interventions within key health areas, pinpointing components that bolster intervention efficacy. Our analysis specifically targeted peer-reviewed research published after the foundational reviews by Head et al [1] in 2013, Hall et al [2] in 2015, and Armanasco et al [3] in 2017. Head et al [1] analyzed 19 studies involving 5958 participants and determined that SMS interventions across various health promotion contexts achieved an average effect size of 0.33 (95% CI 0.27-0.39). A broader examination by Hall et al [2] found a consistent pattern of positive impacts of SMS interventions on diabetes self-management, physical activity, weight loss, smoking cessation, and medication adherence. The assessment by Armanasco et al [3] of the sustained impact of 7 SMS interventions during the postintervention period revealed a modest yet meaningful overall maintenance effect of Cohen *d*=0.17 (95% CI 0.03-0.31), underscoring the enduring benefits of SMS interventions beyond active engagement periods.

### Physical Activity

In 2020, Smith et al [4] conducted a meta-analysis of 10 SMS interventions aimed at enhancing physical activity and found a significant increase in objectively measured steps per day after intervention among participants who received SMS interventions, with an effect size of Cohen *d*=0.38 (95% CI 0.19-0.58). The analysis indicated that interventions incorporating multiple components and tailored messages and those targeted at medical populations tended to yield larger, although not statistically significant, effect sizes. In addition, the study revealed that SMS interventions featuring personalized messages were potentially more effective in promoting physical activity than those using generic text messages (Cohen *d*=0.39 vs 0.25). Similarly, interventions that combined text messaging with other intervention components were more effective than text message–only interventions (Cohen *d*=0.37 vs 0.27).

### Diet and Weight Loss

In 2015, Siopis et al [5] conducted a meta-analysis of 6 studies of SMS interventions targeting weight loss, finding that participants in SMS intervention programs lost significantly more weight (–2.17 kg, 95% CI –3.41 to –0.93) than those in control groups over 8 to 12 weeks. The design of these SMS interventions varied considerably, with intervention lengths ranging from 1 to 24 months and text messaging frequencies varying from daily to biweekly. In 2020, Skinner et al [6] analyzed 12 weight loss interventions involving 1977 participants and observed a mean weight loss difference of –2.28 kg (95% CI –3.17 to –1.36) favoring the SMS intervention groups. Among studies focusing on maintaining weight loss involving 3728 participants, the mean difference in weight change was –0.68 kg (95% CI –1.31 to –0.05) favoring SMS interventions. During postintervention follow-ups, the effect was –0.57 kg (95% CI –1.67 to 0.53), suggesting sustained SMS intervention benefits. These variations in weight loss outcomes were not clearly associated with specific intervention characteristics such as duration, text messaging frequency, theoretical basis, interactivity, or personalization. In 2020, Partridge et al [7] reviewed 8 studies targeting adolescents (767 participants), finding that SMS interventions led to greater reductions in BMI ranging from 1.3% to 4.5% compared to controls by the end of follow-up. These studies exhibited diversity in SMS intervention intensity, duration and content and behavioral strategies used in SMS interventions, underscoring the heterogeneity within intervention research on weight loss and management.

### Mental Health

Within the domain of mental health, most SMS interventions tested focused on depression. In 2019, Senanayake et al [8] conducted a meta-analysis of 7 trials (845 patients; adults: n=664, 78.6%; adolescents: n=181, 21.4%), finding that the standardized mean reduction in depression scores for SMS interventions was 0.23 (95% CI –0.02- to 0.48). In 2020, Cox et al [9] examined 7 SMS interventions (1918 participants) and found, through pooled analysis, nonsignificant differences in depressive symptom scores between the SMS intervention and control groups (mean –0.27, 95% CI –0.54 to 0.01). Reductions in depression were greater when text message content targeted mental well-being and mood improvement and used cognitive behavioral therapy and when the message frequency was ≥2 times per week. Evidence for other mental health issues remains less robustly studied. In 2020, D’Arcey et al [10] performed a systematic review of 15 studies focused on patients with schizophrenia. Although effect sizes were not presented, they commented that most SMS interventions demonstrated positive effects on dimensions of engagement such as medication adherence, clinic attendance, and therapeutic alliance.

### Substance Use

Within the domain of substance use, the most studied behavioral focus for SMS interventions is smoking cessation. In 2016, Scott-Sheldon et al [11] conducted a meta-analysis evaluating the effectiveness of SMS interventions on smoking cessation, analyzing 22 interventions with 15,593 participants from 10 countries. The analysis revealed that individuals who received SMS interventions were significantly more likely to quit smoking compared to control groups, as evidenced by various smoking abstinence measures, including 7-day point prevalence (odds ratio [OR] 1.38, 95% CI 1.22-1.55) and continuous abstinence (OR 1.63, 95% CI 1.19-2.24). In 2023, Zhou et al [12] focused on young adults aged 16 to 30 years and found, across 7 studies, that SMS interventions were effective in promoting 7-day point prevalence abstinence with a risk ratio of 1.83 (95% CI 1.34-2.48).

Following smoking, alcohol misuse is the next most common substance use disorder addressed by SMS interventions. In 2021, Bendtsen et al [13] performed a meta-analysis of 26 trials (7376 participants) examining SMS interventions and found a significant decrease in alcohol consumption among intervention groups compared to controls (Hedges *g*=0.17, 95% CI 0.10-0.23). Subgroup analyses indicated more substantial effects for SMS interventions that used personalized messages (Hedges *g*=0.25), had a high frequency of messaging (Hedges *g*=0.24), and were grounded in behavioral theories (Hedges *g*=0.22).

### Medication Adherence

In 2016, Thakkar et al [14] conducted a meta-analysis of 16 trials testing SMS interventions on medication adherence in chronic disease involving 2742 participants. The findings indicate that SMS interventions significantly enhanced medication adherence (OR 2.11, 95% CI 1.52-2.93). Notably, the effectiveness of SMS interventions did not vary based on the duration of the intervention; the type of chronic disease targeted; or specific features of the text messages, such as personalization, 2-way communication, or the frequency of text messages sent.

Several subpopulations have been the focus of meta-analyses for medication adherence SMS interventions.

For HIV in adolescents, a systematic review by Mehra et al [15] in 2021 included 7 studies with a total of 987 participants, where sample sizes ranged from 14 to 332. Of these 7 studies, 5 (71%) demonstrated a positive impact of SMS interventions in improving adherence. The pooled mean difference between the intervention and the control groups was 0.05 (95% CI –0.08 to 0.17).

For serious mental illness, in 2022, Simon et al [16] analyzed 9 unique trials involving 937 participants. The duration of these studies varied from 30 days to 18 months, with text message frequency ranging from twice a week to 12 times a day. Of these 9 studies, 7 (78%) reported statistically significant improvements in medication adherence and at least 1 clinical outcome.

For vascular disease, in 2017, Adler et al [17] examined 7 randomized trials testing SMS interventions for patients with established arterial occlusive events among 1310 participants. Although heterogeneity precluded meta-analyses, 6 (86%) out of 7 trials showed a significant effect on medication adherence across a range of definitions.

### Summary

This narrative review highlights the substantial and growing evidence base for the effectiveness of SMS interventions across a range of health behaviors and outcomes. Meta-analyses consistently demonstrate small to moderate positive effects of SMS interventions on physical activity, diet and weight loss, mental health, substance use (particularly smoking and alcohol), and medication adherence. There is also emerging evidence for the benefits of SMS interventions in subpopulations such as adolescents and individuals with serious mental illness. However, the optimal design features of SMS interventions (eg, frequency, duration, and personalization) vary across studies and health domains, suggesting the need for further research to identify the most effective intervention components for specific behaviors and populations. In addition, more studies are needed to assess the long-term maintenance of effects and the potential for SMS interventions to help older adults. Despite these limitations, the current evidence supports the effectiveness of the use of SMS interventions in promoting health behavior change and improving clinical outcomes.

## What Makes SMS Interventions Uniquely Useful?

SMS interventions continue to be a powerful tool for health behavior support due to their widespread use and unique functioning as a communication tool. One key reason is that text messaging remains a highly preferred form of communication. In a national sample of 1019 adults across the United States and Canada in 2023, 70% (n=713) of people reported that texting is the fastest way to reach them, 74% read every text they receive, and 62% read texts within a few minutes after receiving them [18]. Another key advantage of text messaging is its efficacy in collecting longitudinal and contextual information from individuals and delivering microdoses of intervention support. Text messaging functions as a “push” technology, delivering messages directly to individuals without any effort on their part [19], which has been shown to be a powerful engagement tool [20]. This contrasts with other digital communication modalities, such as email or web-based messaging, which require users to proactively check for messages. In contrast to text messaging, smartphone app notifications can be turned off, and wearable technologies are not consistently worn [21].

The real-time data collection aspect of SMS interventions provides unique insights into the mechanisms of behavior change. Historically, behavioral intervention studies used sparse measurements taken immediately before and after the delivery of an intervention to examine mediators of distal behavior change [22]. This approach has constrained the ability to identify the dynamic and nonlinear processes through which behavior change likely occurs [23]. Text messaging allows for the low-barrier monitoring of participants, capturing detailed, time-stamped information about their behaviors, emotions, and environmental contexts. This results in a richer data set compared to traditional methods, which often rely on retrospective self-reports prone to recall bias [24]. Dynamic models of behavior change derived from the longitudinal data collected via 2-way SMS interventions so far have begun to offer new insights into behavior change mechanisms [24,25] have begun to offer new insights into behavior change mechanisms.

In summary, the real-time ability to collect information and deliver microdoses of intervention support is the secret superpower of SMS interventions. For researchers, it offers a unique opportunity to explore the intricate time dependencies between various precursors and health behaviors, enhancing our understanding of behavior change mechanisms. For users, it provides immediate, context-relevant support, fostering sustained engagement and promoting long-term health behavior change.

## Key SMS Intervention Design Considerations

### Overview

In this section, we review concepts to consider when designing SMS interventions, focusing on communication strategies, psychological theories, behavior change strategies, and adjunctive functionality. In Table 1 and the sections below, we summarize current best practices based on existing research or personal experience and highlight important open questions and areas where knowledge remains limited or where special consideration should be made. Understanding current best practices for design features and gaps in knowledge is crucial for developers seeking to create effective, engaging, and evidence-based SMS interventions.

**Table 1:** Open questions.

Feature and best practice				Open questions
**Communication Strategies**				
	**Onboarding**			
		Set expectations for the user, including intensity, duration, and what can and what cannot be responded to in real time.	How much baseline information should be captured to allow tailoring and personalization?	
	**Embodiment**			
		Identifying programs with professional affiliation or credible source.	Is there an ethical obligation to remind users they are communicating with an automated computer program?	
	**Personalization**			
		Personalize messages based on individual characteristics, such as name.	Should one use age-specific lingo?	
	**Timing**			
		Determine the optimal timing of messages based on the user’s daily routines and preferences and the target behavior or health condition.	What are the most effective strategies for determining the optimal timing of messages for different health behaviors, user characteristics, and intervention goals?	
		Consider the user’s time zone and sleep schedule when scheduling messages to avoid sending them at inappropriate or disruptive times.	What are the trade-offs between highly personalized timing (eg, based on individual schedules) and more standardized timing (eg, based on general population norms), and how can these be balanced in the intervention design?	
		Allow users to customize or adjust the timing of messages based on their individual needs and preferences.	How can the timing of messages be dynamically adapted based on the user’s changing needs, preferences, and contexts over time?	
	**Intensity**			
		Consider using higher-frequency messaging during critical periods (eg, at the beginning of the intervention or during challenging times).	How can the frequency of messages be personalized based on individual participant characteristics and engagement patterns?	
		Allow participants to adjust the frequency of messages based on their needs and preferences.	What are the best practices for managing message fatigue and maintaining participant engagement over time?	
		Ensure that the frequency of messages does not become overwhelming or lead to message fatigue.	Does allowing voluntary “vacations” from behavioral programs provide long-term engagement benefits?	
		Use data analytics to monitor engagement and adjust frequency accordingly.	Engagement may not always equate to behavioral progress. What techniques can be used to determine the difference?	
	**Duration**			
		Determine the appropriate duration of the intervention based on the complexity of the target behavior, the goals of the intervention, and the needs of the target population.	What is the optimal duration of SMS interventions for different health behaviors, populations, and settings, and how can this be determined empirically?	
		Plan for the gradual tapering or fading of intervention intensity to promote self-sufficiency and prevent relapse.	What are the most effective strategies for promoting the long-term maintenance of behavior change after the initial intervention period?	
		Consider offering booster sessions or periodic check-ins to reinforce skills and maintain motivation after the initial intervention period.	How often should one check in? What exactly should boosters look like?	
**Psychological theories**				
	**General**			
		Select evidence-based psychological theories that are relevant to the target health behavior and population (eg, health belief model, theory of planned behavior, social cognitive theory, and self-determination theory).	What is the optimal way to translate psychological theories into effective SMS interventions?	
		Incorporate key constructs from the selected theories into the intervention design, such as self-efficacy, social support, and motivation.	How can multiple theories be integrated into a coherent intervention design?	
		Consider using a combination of theories to address different aspects of behavior change.	What is the role of emergent theories (eg, JITAIs^a^) in optimizing SMS interventions?	
**Behavior change techniques**				
	**Self-monitoring**			
		Encourage participants to regularly track their health behaviors by providing simple and user-friendly tools for self-monitoring.	How can self-monitoring be made more accessible and engaging for individuals with low health literacy or limited access to technology? How to identify individuals who experience an outsized negative affect due to the increased self-focused attention due to self-monitoring?	
		Tailor the frequency and timing of self-monitoring prompts based on participants’ preferences and adherence patterns.	How can self-monitoring be sustained over the long term, and what are the best strategies for preventing participant burnout?	
		Emphasize the importance of consistent and accurate self-monitoring for behavior change and goal achievement.	What are the most effective ways to integrate self-monitoring data from multiple sources (eg, text messages, wearable devices, electronic health records) to provide comprehensive feedback and support?	
		Consider using gamification techniques to make self-monitoring more engaging and rewarding.	What types of gamification elements (eg, points, badges, leaderboards, challenges, levels) are most likely to resonate with the target audience and what role does social interaction play in the gamification strategy (eg, team-based challenges, peer encouragement)?	
	**Goals setting and action planning**			
		Assist participants in setting SMART^b^ goals aligned with their health behavior objectives.	What is the optimal balance between participant autonomy and intervention guidance in setting and monitoring goals?	
		Break down long-term goals into smaller, actionable steps (ie, action planning) that can be easily monitored and achieved through text message support.	How can SMS interventions be designed to help participants overcome common barriers to goal achievement, such as time constraints, the lack of resources, or competing priorities?	
		Provide regular prompts and reminders to monitor goal progress and encourage participants to report their accomplishments.	What is the role of habit formation and automaticity in sustaining goal-directed behaviors, and how can SMS interventions support these processes?	
		Adapt goals and action plans based on participants’ progress and feedback.	What are the potential unintended consequences of goal-setting and monitoring (eg, feelings of failure or guilt), and how can these be mitigated through text message support?	
	**Feedback**			
		Offer personalized feedback and encouragement based on goal progress, celebrating successes and providing support during setbacks.	How can SMS interventions balance the need for providing regular feedback with the risk of overwhelming or discouraging participants?	
		Provide timely, specific, and actionable feedback on participants’ reported behaviors, such as progress toward goals, adherence to recommended actions, or trends over time.	What is the role of machine learning and adaptive algorithms in generating personalized and context-aware feedback messages?	
		Use a combination of positive reinforcement (eg, praise for successes) and constructive feedback (eg, suggestions for improvement) to maintain motivation and engagement.	How can the effectiveness of different feedback strategies be evaluated and compared across SMS intervention designs and health behavior domains?	
		Offer feedback in a nonjudgmental and supportive tone, emphasizing the participant’s efforts and progress.	What are the potential unintended consequences of providing feedback on behavior (eg, increased anxiety or self-criticism), and how can these be mitigated through supportive messaging and resources?	
**Functionality**				
	**Human helpers**			
		Identify key social support networks and human resources that can enhance the effectiveness of text messaging interventions, such as family members, health care providers, peer support groups, and research staff.	What are the optimal roles and responsibilities of different types of human helpers in SMS supporting text messaging interventions, and how can these be defined and communicated effectively?	
		Use text messaging to facilitate communication and coordination between users and their human helpers, such as sharing progress updates, requesting support, or scheduling appointments.	How can human helpers be trained and motivated to provide high-quality, consistent, and empathetic support to users, particularly over long periods?	
		Tailor the involvement of human helpers to the user’s individual needs, preferences, and social context, and allow for flexibility and adaptability over time.	What are the potential risks and challenges of involving human helpers in text messaging interventions, such as breaches of confidentiality, boundary violations, or inconsistent support, and how can these be mitigated?	
	**Multiplatform**			
		Consider integrating text messaging with other digital health platforms, such as web pages or mobile apps, to provide a comprehensive and consistent user experience.	How can data from multiple platforms be effectively integrated and analyzed to provide a holistic view of user engagement and progress?	
		Ensure that the text messaging component complements and enhances the functionality of other platforms, rather than duplicating or conflicting with them.	What are the technical, logistical, and financial challenges of developing and maintaining a multiplatform intervention, and how can these be addressed?	
		Provide clear guidance to users on how to navigate between different platforms and access the full range of intervention features.	How to best assess engagement across various digital platforms?	
	**Sensor integration**			
		Explore opportunities to integrate text messaging with data from body-worn sensors (eg, Fitbits [Fitbit Inc]), ambient sensors (eg, sensors in the home or car), and connected devices (eg, Alexa [Amazon.com, Inc]) to provide more personalized and context-aware support,	What types of sensors and connected devices are the most effective and acceptable for different health behaviors and populations, and how can these be seamlessly integrated with text messaging?	
		Use sensor data to trigger JITAIs delivered via text message based on the user’s current state and context.	How can sensor data be used to develop more precise and personalized models of behavior change, and what are the implications for intervention design?	
		Ensure that the integration of sensor data is transparent, secure, and aligned with user preferences and expectations.	How can the validity, reliability, and meaningfulness of sensor data be ensured, and what are the implications for intervention fidelity and effectiveness?	

^a^JITAI: just-in-time adaptive intervention.

^b^SMART: specific, measurable, achievable, relevant, and time-bound.

### Communication Strategies

#### Overview

One key reason why SMS interventions are so effective for relationship building is their ability to simulate a human-like dialogue. The way in which information is conveyed, the tone of the messages, and the timing and frequency of delivery can all have a significant impact on users’ engagement; motivation; and, ultimately, behavior change outcomes. This section explores several key communication strategies in SMS intervention design, including onboarding, embodiment, personalization, timing, intensity, and duration. By examining current best practices and identifying open questions in each of these areas, we aim to shed light on the critical role of communication in creating engaging, persuasive, and effective SMS interventions.

#### Onboarding

Clear communication during the onboarding process can help set expectations, build rapport, and increase motivation for engagement with the SMS intervention. Defining expectations is the first critical design component of a well-functioning SMS intervention and should include, at minimum, conveying information about the SMS intervention’s intensity and duration. Additional information captured during the onboarding process could be used to personalize the material.

#### Embodiment

Identifying the program as originating from a credible source can enhance trust and engagement [26]. Regarding transparency, a major open question is whether and how SMS intervention designers should disclose that the program is automated [27], explicitly reminding users that there is no human provenance when there is none or articulating what the limits of human involvement are.

#### Personalization

Personalizing messages with user characteristics such as name is likely to contribute to engagement [28]. By using natural language, SMS interventions can create a sense of warmth, empathy, and personalization that resonates with users. Beyond this, it remains unknown as to how personalization such as the use of cohort-specific identifiers impacts engagement and effectiveness. It is, however, crucial for SMS interventions to avoid labeling someone as having a disease or condition [29], which can lead to the internalization of negative stereotypes and self-stigma, which can erode self-esteem and the motivation to engage in healthy behaviors [30].

#### Timing

For many behaviors, knowing when to interact with users may be as crucial as knowing what to do. Research on habit formation and context-dependent learning [31] suggests that behaviors are strongly influenced by the contexts and cues in which they occur. By delivering interventions at specific moments and in specific contexts, it may be possible to interrupt impending undesired habits or promote the formation of positive habits. However, identifying when these critical moments are likely to occur presents a significant challenge. Some behaviors may exhibit predictable patterns at the population level, such as increased sedentary behavior during the workday or decreased medication adherence on weekends. Other behaviors, however, may show high within-person variability, making it more challenging to predict when an individual will need support. Depending on the behavior and the necessity to control the timing of messages, consideration could be made to allow users to set their own preferred times for receiving messages.

#### Intensity (Dose)

The key to optimizing SMS intervention intensity is to provide sufficient support and guidance to promote behavior change while avoiding overwhelming or burdening the individual. The optimal messaging frequency may vary depending on the nature of the target behavior and its temporal dynamics. For behaviors that occur at short intervals, such as smoking or snacking, more frequent interactions may be necessary to provide timely support and reinforcement. In contrast, for behaviors that occur at longer time scales, such as physical activity or medication adherence, a more spaced-out cadence of messaging may be appropriate. However, it is important to recognize that simply increasing the intensity of messaging, particularly for challenging or complex behaviors, may not always lead to better outcomes [32]. In fact, this approach could backfire by overwhelming or frustrating users, leading to disengagement or even reactance [33]. An open question is how to taper the frequency or intensity of messages over time to gradually transfer the responsibility for behavior change from the intervention to the individual. Another related question is how often, for frequent behaviors, one must interact to be effective.

#### Duration

As with all behavioral interventions, key considerations for SMS interventions include how long they must run to be effective. Research suggests that it takes an average of 66 days for a new behavior to become a habit, but this can vary widely depending on the complexity of the behavior and individual differences [34]. There is also evidence that the duration of engagement with SMS interventions does not necessarily translate to more favorable outcomes [35,36]. In our experience, the relationship between SMS intervention engagement and effectiveness is U-shaped: individuals who show the greatest effects either use the program the least (indicating they have adopted the behavior and do not need the support) or the most (indicating their commitment to the work of behavior change). A fixed duration may be appropriate for interventions targeting short-term goals or behaviors with a clear end point, such as completing a vaccination schedule or recovering from surgery. However, for interventions targeting complex, long-term behavior changes, such as adopting a healthy diet or increasing physical activity, a more flexible and individualized approach may be necessary.

### Psychological Theories

The incorporation of psychological theories into the design of SMS interventions has been a topic of growing interest. The meta-analysis by Head et al [1] and the systematic review by Hall et al [2] both found that SMS interventions based on theories were significantly more effective in promoting health behaviors than those not based on theories. Several specific theories have been commonly used in the design of SMS interventions. The transtheoretical model [37], which posits that individuals move through distinct stages of change when modifying health behaviors, has been successfully applied in SMS interventions for physical activity and smoking cessation [38]. Social cognitive theory [39], which emphasizes the interplay among personal, behavioral, and environmental factors in shaping behavior, has also been frequently used in SMS interventions for various health behaviors [38]. Other theories that have informed SMS intervention design include the health belief model [40], which focuses on individuals’ perceptions of health risks and benefits, and the theory of planned behavior [41], which emphasizes the role of attitudes, subjective norms, and perceived behavioral control in shaping intentions and behaviors [42].

While evidence supports the use of these traditional theories in SMS intervention design, some researchers have argued that existing theories may need to be adapted or integrated to better suit the unique context of mobile interventions [43]. For example, existing theories could be updated and adapted to take into account the dynamic and interactive nature in which behaviors play out in the real world, as well as the potential for real-time interventions to adapt to those time- and context-dependent needs [44]. For example, mathematical equations informed by control theory [45] could be used to make predictions at a given time point for a given individual based on prior data from the individual to tailor some aspect of intervention content. Testing of such dynamical models is in the early stages [46,47]; thus, it remains unknown whether the complexity pays off in requisite effects.

### BCTs Used in SMS Interventions

Self-monitoring, goal-setting, action planning, and feedback are 3 key BCTs that have been widely incorporated into behavioral interventions in general [22,48] and digital interventions (including SMS interventions) specifically [49].

#### Self-Monitoring

Many behaviors, especially when they become habitual, are below the level of conscious recognition [50]. The act of self-monitoring itself can raise awareness of one’s behavior and motivate change. SMS interventions offer a convenient and accessible platform for prompting and assisting individuals in bringing behaviors, emotions, and desires to awareness. This can involve prompting individuals to reflect before a given behavior (to raise awareness of motives and expectancies) or following a behavior (to promote active reflection). It can also have varying levels of specificity, from simply prompting a reflection on a behavior (eg, “Think about how exercising made you feel?”) to requesting specific data (eg, “Please reply with the number of steps you took today.”). The frequency and format of self-monitoring prompts should be tailored to individual preferences and needs weighed against program requirements for tailoring. An open question remains how to identify individuals who experience an outsized negative affect due to the increased self-focused attention due to self-monitoring [51].

#### Goal-Setting and Action Planning

*Goal-setting* and *action planning* are closely related BCTs that assist behavior change [52]. A meta-analysis of 141 studies testing 384 effect sizes (16,523 participants) found a positive unique effect of goal-setting across a range of behaviors (Cohen *d*=0.34, 95% CI 0.28-0.41) [53]. SMS interventions should ideally be designed with specific, measurable, achievable, relevant, and time-bound goals [31]. Relatedly, action planning should ideally involve the concept of implementation intentions: the more specific someone can be with *when, where,* and *how* a goal will be achieved, the more likely it is to be accomplished [54]. SMS interventions can provide templates or examples to help users develop effective goals and action plans and can send reminders or prompts to encourage adherence to the plans. An open question remains how best to support autonomy in self-determined goals [55] while pushing individuals who may not be intrinsically motivated toward progress.

#### Performance Feedback

The principles of cybernetics and control theory [45] suggest that feedback operates as part of a closed-loop system, wherein individuals compare their current behavior to their goals and adjust their actions accordingly. SMS interventions can leverage this feedback loop by providing regular, timely feedback and encouraging users to reflect on their progress and adjust as needed. Feedback can help individuals gauge their progress, identify areas for improvement, and adjust their strategies as needed. In SMS interventions, behavioral feedback can be descriptive (eg, “You walked an average of 8,000 steps per day this week.”) or evaluative (eg, “Great job! You exceeded your goal of 7,000 steps per day this week!”). The frequency, tone, and format of feedback messages can be tailored to individual preferences and needs to maximize engagement and effectiveness. One open question is how best to balance the need for providing regular feedback with the risk of overwhelming or discouraging participants. Another open question is how to avoid behavioral disengagement [56] when an individual exhibits persistent goal failures.

### Enhanced Functionalities

#### Human Helpers

Automated systems for delivering SMS interventions offer several advantages, including scalability and reproducibility. However, a key limitation of automated SMS interventions is their inability to provide supportive accountability, which can be crucial for patient engagement and support [57]. To address this, researchers have explored incorporating human helpers, such as health care professionals, significant others, and peers, into SMS interventions.

One early example of incorporating professionals into SMS interventions was a study by Rodgers et al [58], which evaluated a smoking cessation program where participants received personalized text messages from a team of counselors. The counselors used a web-based interface to monitor participant progress and tailor the content and timing of messages based on individual needs. To capitalize on the strengths of both human and automated approaches, some SMS interventions have adopted a hybrid model. Aguilera et al [59] evaluated a hybrid SMS intervention for depression that used an automated system to send daily mood-tracking prompts and self-management tips while also allowing participants to request additional support from a mental health coach via an text message. This hybrid approach aimed to strike a balance between the efficiency of automation and the benefits of human interaction.

Dyadic SMS interventions, which involve significant others, have been studied primarily in adolescent populations, addressing conditions such as asthma [60], diabetes [61], and mental health conditions [62]. In adult populations, dyadic SMS interventions have been explored for promoting physical activity [63]. Best practices for implementing dyadic SMS interventions include identifying key social support networks, providing training and guidance to human helpers, facilitating communication between users and helpers via text messaging, and tailoring the involvement of helpers to individual needs and preferences. However, several open questions remain regarding the optimal roles and responsibilities of different types of human helpers; strategies for ensuring the quality and consistency of support; and approaches to addressing potential risks and challenges, such as breaches of confidentiality or inconsistent support. Future research should aim to address these questions to further optimize the integration of human support into SMS interventions.

#### Multiplatform Integration

There is evidence that using multiple modes of digital communication (ie, combining text messaging with other digital communication platforms, such as web pages or mobile apps) can enhance health behavior change [64]. Best practices include ensuring that the text messaging component complements and enhances the functionality of other platforms, providing clear guidance on navigating between platforms, and using data from multiple sources to inform personalization. For example, there are many self-report behavioral instruments composed of more than a few questions that would be tedious if deployed through text messaging. In addition, there are forms of feedback such as interactive graphs that require other digital modalities. There remain open questions around the optimal combination and sequencing of communication channels, the challenges of developing and maintaining multiplatform interventions that can communicate with one another, and challenges of determining which components are necessary for effectiveness.

#### Integration With Sensors and Connected Devices

Integrating text messaging with data from body-worn and ambient sensors presents an opportunity to deliver more personalized and context-aware support. For example, studies have found promising results when using SMS interventions integrated with activity monitors [65], weight scales [66], physiological monitors (eg, blood glucose [67] and blood pressure [68]), and location-monitoring technology (GPS) [69]. It is certain that the use of wearable sensors [70] and ambient environmental sensors [71] will play a larger role in health monitoring and behavioral interventions. Open questions remain regarding the most effective types of sensors for different health behaviors, the level of certainty needed for inferred behaviors, the development of precise and personalized behavior change models, the optimal algorithms for triggering interventions or support, and the ethical and social implications of using sensor data.

## Future Opportunities: LLMs as SMS Intervention Conversational Agents

### Overview

To date, SMS interventions have primarily used rule-based systems that process simple client inputs through branching algorithms to generate prewritten text responses. For example, a system might compare a reported alcohol consumption quantity against a preset threshold to trigger specific messages about drinking behavior. Although efforts to enhance interactivity through conversational agents such as chatbots have shown promise in promoting behavior change across various domains [72], including mental health [73] and substance use [74,75], these systems often lack the adaptability required for deep therapeutic engagement.

### LLMs as Therapeutic Conversational Agents

The advent of LLMs [76] marks a potentially transformative advancement in conducting automated therapeutic conversations. LLMs, such as ChatGPT (OpenAI), are deep learning models trained on extensive textual data capable of understanding the context and relationships between words and generating responsive, novel text that is coherent and contextually appropriate [77]. These models can be fine-tuned after training for specific tasks or domains, enhancing their adaptability for diverse applications [78], including psychotherapy [79]. Emerging studies highlight LLMs’ capabilities in areas such as emotional awareness [80], social awareness [81], empathy [82], creativity [83], and reasoning [84]. Preliminary findings suggest that ChatGPT can engage positively in therapeutic conversations [85], actively listen, provide validation, and suggest coping strategies [86]. Trials comparing rule-based and generative counseling are still in early stages [87].

### Training LLMs

When developing LLM-based SMS interventions for health behavior support, researchers are advised to prioritize pretraining with domain-specific data sets to guarantee that the LLM produces accurate and relevant responses. This essential step involves compiling a diverse data set, including successful SMS interventions’ text libraries, contributions from human experts, counseling manuals, and psychological theories. These types of data sets should be used to familiarize the LLM with effective language patterns, intervention strategies, and the appropriate tone for health behavior change. The training process should be carefully customized to stress the significance of clarity, empathy, and motivational encouragement, which are crucial for impactful health communication. In addition, the integration of both preformed and real-time responses is crucial, as real-time LLM-generated content can offer a dynamic conversational experience, while preformed replies ensure consistency and accuracy for sensitive topics.

### Addressing the Unpredictability of LLMs

Due to their generative nature, LLM-based conversations can vary in quality and appropriateness. We suggest several key strategies to minimize untoward communication events. The first line of defense involves *LLM guardrails* [88], custom configurations within the LLM that prevent the generation of harmful or inappropriate content, including filters for sensitive topics and checks for alignment with therapeutic best practices. The second strategy is to incorporate a *human-in-the-loop system* [89], where a trained research team member reviews all messages and can edit and clarify any undesired message. These techniques ensure that LLM-based SMS interventions are both effective in promoting health behaviors and in conformance with high standards of quality and ethical practice.

## Future Challenges: Bridging SMS Interventions From Research to Real-World Application

Currently, several SMS interventions are available to the US public, offering evidence-based support across various health domains. Notable examples include Text4baby, which provides maternal health information; VEText, which offers appointment reminders to US veterans; and Smokefree, which offers smoking cessation support. While these programs demonstrate the efficacy of SMS interventions, they are primarily accessible through government and foundation channels, underscoring the need for enhanced and centralized dissemination methods.

SMS interventions face several key challenges in transitioning from research environments to widespread real-world implementation and effectiveness. Unlike apps, which benefit from commercial platforms such as app stores facilitating consumer access and enabling monetization, SMS interventions lack a marketplace. This gap restricts commercial innovation and the potential for broader adoption. Furthermore, health systems, ideally positioned to leverage SMS interventions for enhancing patient engagement, encounter barriers due to outdated legal apprehensions regarding the security of text message communication. Concerns about the potential for unauthorized access to sensitive patient data and the technical difficulties of integrating SMS functionalities with existing health informatics systems further complicate implementation efforts, often requiring significant technological investment. Finally, there is the concern that outside the context of a controlled trial, SMS intervention effectiveness will be hampered by low longitudinal user engagement.

To address these challenges and foster the adoption of SMS interventions, several strategies are proposed. First, establishing a dedicated commercial portal for SMS interventions could mirror the app store model, offering a platform for users to access evidence-based interventions. This would not only enhance visibility but also stimulate commercial interest and development. Second, adopting standardized informed consent procedures would enable patients to opt into SMS interventions knowingly and willingly, mitigating legal and ethical concerns. In addition, formulating comprehensive policies and guidelines for digital communication within health systems can promote consistency, compliance, and confidence in the security of text message exchanges. Examples of health systems successfully implementing SMS interventions in routine health care are, however, growing. For example, Bressman et al [90] reported on the implementation of a 30-day postdischarge intervention using automated texting to supplement the standard of care within a single primary care practice in Penn Medicine. They found that the adjusted OR for an emergency department visit was 0.77 (95% CI 0.45-1.30) and for a readmission was 0.45 (95% CI 0.23-0.86). Educating health care providers on the value and utility of SMS interventions could prompt them to prescribe these interventions as part of a treatment plan, much like any conventional medication. Finally, we believe that engagement with SMS interventions can be optimized through thoughtful design focused on matching user needs with support provision. For example, we have found that for SMS interventions of longer duration, voluntary vacations or breaks from SMS interventions could reduce attrition [91] and accommodate self-determination [92].

## Conclusions

In this paper, we have attempted to capture the essence of SMS interventions, recognizing their fundamental yet often underestimated role in shaping health behaviors. Their apparent simplicity masks a capacity for delivering health interventions that are both nuanced and highly personalized, effectively transmitting information, offering support, and bridging gaps in health care services. The true power of text messaging emerges through its steady and subtle influence over time rather than through immediate, dramatic effects.

From the initial tests in the early 2000s to a peak of scholarly activity in the early 2020s, SMS interventions have demonstrated a broad spectrum of application across critical health behaviors and outcomes, underscored by a steady growth in both academic interest and funding. The narrative review further solidifies the evidence base for SMS interventions, showcasing their effectiveness in promoting physical activity, aiding weight loss, supporting mental health, reducing substance use, and enhancing medication adherence, with nuanced effects across diverse populations and health domains. We posit that this broad and consistent effectiveness is largely due to the ongoing widespread use of text messaging to communicate and unique functioning of text messaging as a communication tool.

The exploration of SMS interventions’ key features reveals a delicate balance between technological advancement and human-centric design principles, underscoring the importance of personalization, timing, intensity, and the integration of psychological theories. These considerations are pivotal in designing SMS interventions that not only engage users but also instigate meaningful behavioral change. The emergence of generative artificial intelligence presents an exciting frontier, promising to enrich SMS interventions with more natural and adaptive conversational capabilities, provided that ethical standards and quality assurance mechanisms are rigorously maintained.

Despite all this support and promise, the commercialization of SMS interventions, their integration into health care systems, and their engagement outside controlled studies remain ongoing challenges. However, the potential of SMS interventions to bridge the communication gap between patients and providers is immense. With a strategic emphasis on developing a marketplace, patient consent procedures, policy development, medical education, and optimizing user engagement, SMS interventions could revolutionize health care delivery, enhancing patient engagement and support across the continuum of care.

As we navigate the future of health communication, the insights garnered from 2 decades of SMS intervention research illuminate the path forward, highlighting the need for continued innovation, integration, and interdisciplinary collaboration. As different messaging platforms arise, such as WhatsApp (Meta Platforms, Inc), Facebook Messenger (Meta Platforms, Inc), WeChat (Tencent Inc), and Snapchat (Snap Inc), existing evidence from SMS interventions can inform design. By harnessing the simplicity, adaptability, and depth of SMS interventions, we can continue to shape the landscapes of health behavior and support, making strides toward improved health outcomes and reduced disparities in the years to come.

## Appendix

Multimedia Appendix 1 PubMed search terms.

## Abbreviations

BCT: behavior change technique
LLM: large language model
OR: odds ratio
