# Supplementary material for: Deceptively Simple yet Profoundly Impactful: Text Messaging Interventions to Support Health
Source: J Med Internet Res. 2024 Aug 27;26:e58726. doi: 10.2196/58726 (PMC11387917; doi:10.2196/58726)
Supplement: Multimedia Appendix 1 [file jmir_v26i1e58726_app1.docx]

**Multimedia Appendix 1**

PubMed search terms:

1. Physical activity: ("Text Messaging"[Mesh] OR "text messag*" OR "SMS" OR "short message service" OR "text-based" OR "texting") AND "text"[Ti] AND (intervention* OR trial OR study) AND ("physical activity" OR "physical activities" OR exercise* OR "physical fitness" OR "physical endurance" OR "physical exertion" OR "physical performance" OR "physically active" OR "activity") AND "activity"[Ti]
2. Diet & Weight Loss: Text Messaging[Mesh] OR "text messag*" OR "SMS" OR "short message service" OR "text-based" OR "texting") AND "text"[Ti] AND (intervention* OR trial OR study) AND ("diet"[Ti] OR "weight"[Ti])
3. Mental Health: "Text Messaging"[Mesh] OR "text messag*" OR "SMS" OR "short message service" OR "text-based" OR "texting") AND "text"[Ti] AND (intervention* OR trial OR study) AND ("mental"[Ti] OR "depress*"[Ti] OR "anxiety"[Ti] OR "schiz*"[Ti] OR "bipolar"[Ti] OR "suicid*"[Ti])
4. Substance Use: "Text Messaging"[Mesh] OR "text messag*" OR "SMS" OR "short message service" OR "text-based" OR "texting") AND "text"[Ti] AND (intervention* OR trial OR study) AND ("alcohol"[Ti] OR "substance"[Ti] OR "cannabis"[Ti] OR "cocaine"[Ti] OR "cigare*"[Ti] OR "tobacco*"[Ti] OR "smok*"[Ti] OR "opioid"[Ti] OR "heroin"[Ti]"))
5. "Text Messaging"[Mesh] OR "text messag*" OR "SMS" OR "short message service" OR "text-based" OR "texting") AND "text"[Ti] AND (intervention* OR trial OR study) AND ("medication"[Ti] OR "disease"[Ti] OR "diabet*"[Ti] OR "hypertension"[Ti] OR "heart"[Ti] OR "asthma"[Ti] OR "arthritis"[Ti] OR "skin"[Ti] OR "pain"[Ti] OR "HIV"[Ti] OR "kidney"[Ti] OR "heart"[Ti] OR "stroke"[Ti] OR "COPD"[Ti] OR "cancer"[Ti] OR "liver"[Ti] OR "Parkins*"[Ti] OR "dement*[Ti])))
6. Reproductive & Maternal Health: ("Text Messaging"[Mesh] OR "text messag*" OR "SMS" OR "short message service" OR "text-based" OR "texting") AND "text"[Ti] AND (intervention* OR trial OR study) AND ("maternal health"[Ti] OR "reproductive health"[Ti] OR "breastfeed*"[Ti] OR "breast feed*"[Ti] OR "infant feeding"[Ti] OR "lactation"[Ti] OR "condom*"[Ti] OR "contraceptive*"[Ti] OR "birth control"[Ti] OR "family planning"[Ti] OR "safe sex"[Ti] OR "safer sex"[Ti] OR "sexual health"[Ti] OR "sexually transmitted"[Ti] OR "STI"[Ti] OR "STD"[Ti] OR "HIV"[Ti] OR "AIDS"[Ti] OR "pregnancy"[Ti] OR "prenatal"[Ti] OR "antenatal"[Ti] OR "postnatal"[Ti] OR "postpartum"[Ti] OR "obstetric*"[Ti] OR "gynecolog*"[Ti] OR "women's health"[Ti])
7. Other: ("Text Messaging"[Mesh] OR "text messag*" OR "SMS" OR "short message service" OR "text-based" OR "texting") AND "text"[Ti] AND (intervention* OR trial OR study)
